# Enhancing the Quality of Ready‐to‐Eat Trout Burger Patties: Investigating the Combination of Modified Atmosphere Packaging and Microbial Transglutaminase Enzyme

**DOI:** 10.1002/fsn3.4495

**Published:** 2024-10-01

**Authors:** Can Okan Altan, Hülya Turan, Demet Kocatepe, İrfan Keskin, Bayram Köstekli

**Affiliations:** ^1^ Department of Seafood Processing Technology, Faculty of Fisheries Sinop University Sinop Türkiye

**Keywords:** burger, microbial transglutaminase, modified atmosphere packaging (MAP), ready‐to‐eat product, texture

## Abstract

In this study, rainbow trout patties (*Oncorhynchus mykiss* Walbaum, 1792) were treated under a modified atmosphere (MA) (60/40:CO_2_/N_2_) packed with microbial transglutaminase enzyme (MTGase) at varying concentrations (0.5% and 1%) and kept for a full day at 2°C ± 2°C. Denser and more complex intermolecular cross‐linking formations between peptides and gel network structures were observed in the first and last scanning electron microscope images of MTGase‐containing groups. MTGase increased the springiness, hardness, shear force, and work‐of‐shear parameters during the storage period in uncooked and cooked patties compared to the control group. The protein, energy, moisture, and amino acid contents were maintained in the MTGase‐containing groups, but using MTGase at higher concentrations was ineffective. Microbiological analyses have shown that MTGase is partially effective on microbial fauna, and the most significant inhibitory effect was determined on total mesophilic aerobic bacteria (TMAB). In all groups, the total amounts of saturated, monosaturated, and polyunsaturated fatty acids remained unchanged on both production and expiration days (*p* > 0.05). After mixing the burger patty additives into the ground meat, the amounts of sodium, calcium, potassium, and magnesium increased in all groups (*p* < 0.05); however, a decrease in calcium was observed in the MTGase‐containing groups (*p* < 0.05) compared to the control group. The higher proportion of the MTGase‐contained group did not exhibit significant differences compared to the lower proportion of the MTGase‐contained group, except for textural characteristics and the TMAB inhibition effect.

## Graphical abstract

In this study, rainbow trout patties (*Oncorhynchus mykiss* Walbaum, 1792) were treated under a modified atmosphere (MAP) (60/40:CO_2_/N_2_) packed with microbial transglutaminase enzyme (MTGase) at varying concentrations (0.5% and 1%) and kept for a full day at 2°C ± 2°C.

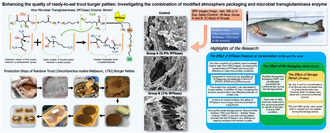

## 1 Introduction

Nutrition habits, a critical factor in human life, have undergone significant transformations in parallel with the innovations and developments in social life from the past to the present. These changes are not merely a reflection of societal progress but are intricately linked to technological advancements, globalization, and shifts in consumer awareness and preferences (Popkin, Corvalan, and Grummer‐Strawn 2020). The evolution of dietary patterns has been particularly pronounced in recent decades, with a noticeable trend toward more processed foods, increased protein consumption, and a growing interest in functional foods (Kearney 2010; Popkin, Corvalan, and Grummer‐Strawn 2020). One of the essential commercial purposes of the food industry is to develop new products with different flavors, appearances, and textural features to align with the evolving nutritional habits and preferences of consumers (Cardoso, Mendes, and Nunes 2007; Gharibzahedi et al. 2018). This urge for innovation is not only a response to changing consumer demands but also a strategy to maintain competitiveness in a rapidly evolving market. Asioli et al. (2017) highlighted that recent studies have increased the importance of sensory attributes, convenience, and perceived healthiness in consumer food choices. In the realm of meat processing, significant advancements have been made in the restructuring. Restructuring meats allows the production of new flavors, better appearance, shape, and desired textural properties. Simultaneously, in parallel with current research development, chemical use and raw material waste can be minimized (Gharibzahedi et al. 2018). Moreover, this approach is well aligned with the increasing consumer demand for more sustainably and efficiently produced food products (Hocquette et al. 2018).

When planning industrial‐scale production of restructured products, it is highly common to prioritize fish species readily available in abundance and cost‐effective for processing. The preferred fish species naturally varies by region, as each geographical area may have access to species at advantageous prices. In the context of Türkiye, an assessment of the situation reveals that large‐sized rainbow trout are cultivated in substantial quantities in the Black Sea Region and inland waters (Turkish Statistical Institute 2023). These cultivated trout present a highly suitable option for aquaculture processing facilities in terms of cost‐effectiveness and year‐round availability. In this study, the most significant factors for preferring the large rainbow trout include ease of regional supply, availability in almost all seasons, and high nutritional value. These characteristics make large rainbow trout an ideal candidate for industrial‐scale production of restructured fish products in the Turkish aquaculture sector.

The market's most widely known enzymatic restructuring agent is transglutaminase (TG), which catalyzes covalent cross‐linking between proteins (De Jong and Koppelman 2002). Microbial transglutaminase (MTGase) is a form of TG produced by microorganisms, usually from the bacterium *Streptomyces mobaraensis* (Motoki and Seguro 1998). MTGase is much more adaptable for use on a commercial scale. In the same way, as in TG, MTGase is used to modify and improve foods' functional properties by catalyzing the formation of intra‐ and intermolecular cross‐links between proteins or peptides (Gaspar and Goes‐Favoni 2015). MTGase enzyme is very effective in terms of forming gel structures in low‐viscosity (fluidity) protein solutions and/or dispersions (sprinkle), forming the mechanical structures in low‐fat proteins, increasing the mechanical strength, providing the missing amino acid addition to the product, and reducing the textural deformation (Motoki and Seguro 1998). In addition, MTGase has a beneficial effect on preserving precious polyunsaturated fatty acids (PUFAs) in seafood, which are very sensitive to oxidation (Pourashouri et al. 2014). Various researchers have reported that the utilization of MTGase in products such as fish balls, fish burger patties, and minced fish results in increased cooking yield and sensory scores, allows for a firmer texture, extends shelf life, increases brightness values, reduces lipid oxidation, and significantly inhibits microbiological load, thereby enhancing overall quality (Huang and Clarke 2017; Altan 2020; Altan et al. 2023). Moreover, MTGase in high‐protein foods could help manage energy intake and reduce the risk of food allergies (Xing et al. 2016). In addition to all these advantages, MTGase's most highlighted benefits are that it does not require the use of chemicals such as emulsifiers in foods, it has a more positive perception for consumers, and its use in foods is classified as “Generally Recognized as Safe (GRAS)” by the U.S. Food and Drug Administration (2022).

Modified atmosphere packaging (MAP) has become a standard method for delaying spoilage in fresh, frozen, and chilled foods and extending the shelf life of fresh aquatic products (Ashie and Lanier 2000). There are many advantages of packaging in a MAP, such as increasing the shelf life of the product by 50%–400%, reducing the economic losses, enabling it to be transported to longer distances, reducing distribution costs, minimizing losses, and so providing more durable products (Devlieghere, Gil, and Debevere 2002). Many studies have demonstrated that the MAP method not only substantially inhibits the aerobic bacteria of seafood but also keeps the sensory and chemical quality as high as possible (Ashie and Lanier 2000; Sivertsvik, Jeksrud, and Rosnes 2002; Del Nobile et al. 2009; Kocatepe et al. 2016; Babic Milijasevic et al. 2023). This study aimed to determine the combined effect of MAP and MTGase on trout burger patties' nutritional, structural, and nanostructural aspects.

## 2 Materials and Methods

### 2.1 Materials

In total, 55 rainbow trout (*Oncorhynchus mykiss* Walbaum, 1792) were used in the research. The fish were obtained from Kuzey Fisheries Comp. (Samsun, Türkiye). The average weight, length, and pH of fish were determined as 1669.60 ± 99.40 g, 44.93 ± 0.93 cm, and 6.78, respectively. All fish were harvested at the facility using the hypothermia method and packaged in polystyrene boxes at a fish/ice (w/w) ratio of 1:3. Afterward, all fish were gutted, filleted, deboned, and skinned. All the fillets were washed under cold tap water and kept in the iced water for 30 min to remove any remaining blood, scales, or impurities. Then, the fillets were left in a stainless‐steel strainer to be drained.

Ingredients such as onions, tomato/pepper paste, dried bread powder, etc. were exposed to 55‐watt ultraviolet light at 50 cm for 1 h to reduce the microbial load. Activa GS‐EU (powder form in 100 g packs; 60 U/g activity) (Ajinomoto Corp., Tokyo, Japan) brand MTGase enzyme was used in the research. Distilled water was used to solubilize MTGase (MTGase:distilled water [g:mL] ratio of 1:4).

### 2.2 Methods

#### 2.2.1 Preparation of Burger Patties and Groups

The fillets were minced with a 2 mm outlet hole meat grinder. The minced fish meat was weighed and determined to be 46.80 kg and divided into three parts (15.60 kg). The production steps were completed, and the groups are shown below.

The ingredients used for the trout patties were as follows: 5% sunflower oil, 1.5% salt, 0.1% garlic powder, 1% sweet pepper paste, 0.25% black pepper, 0.5% cumin, 0.5% tomato paste, 0.25% mixed flake pepper, 5% onion, 5% fine white flour.

Control: 15.60 kg of minced fish meat was homogenized with ingredients in the cutter machine for 3 min at 5000 rpm.

Group A (0.5% MTGase): 15.60 kg of minced fish meat was homogenized with ingredients for 2 min at 5000 rpm in the cutter, and 0.5% MTGase enzyme was added to the mixture, and homogenization was continued for one more minute.

Group B (1% MTGase): 15.60 kg of minced fish meat was homogenized with ingredients for 2 min at 5000 rpm in the cutter, and 1% MTGase enzyme was added to the mixture, and homogenization was continued for one more minute.

Patties were shaped with a hamburger mold (*r* = 7.25; *h* = 0.90 cm; *w* = 150 ± 2 g). A total of 372 burger patties were produced. Industrial hydrophilic pads were placed on Styrofoam plates (160 × 320 × 32 mm) underneath the patties as water retainers. Two burger patties were placed on each plate (186 packages). Before MAP, the average temperature in the center of the patty was measured and found to be 6°C ± 0.2°C. All the patties on the plates were packaged with a MAP at a ratio of 60/40 (CO_2_/N_2_). Plastic MAP packages' thickness, water vapor, and oxygen permeability were 98 μm, 3.48 g/m^2^/day, and 47.60 mL/m^2^/day, respectively. In the package, burger patty and gas mixture ratios were adjusted volumetrically as 1/3 (v/v). Before the storage phase, all packages were kept at 5°C ± 2°C for 6 h due to superior enzyme activation. After that, all packages were stored in the refrigerator at 2°C ± 2°C for 24 days.

#### 2.2.2 Sampling and Analyzing Periods of Burger Patties

The 62 packages of burger patties were produced for each group, and packages were divided into two parts (31 packages) and evaluated as replicates of the groups. Two packages were randomly taken from each group's packages on the sampling days. Proximate composition, amino acids, fatty acids, and mineral content analyses were performed on raw fish and all groups on the first day (day‐0) and spoilage dates. Scanning electron microscope (SEM) images of all groups were taken on production and expiration days. Texture Profile Analysis (TPA), Warnes Bratzler Shear (WBS) (*n* = 12), in‐pack gas measurements (*n* = 4), and microbiological analyses (*n* = 6) were conducted every third day, both in uncooked and cooked patties, until the end of the research (24 days).

#### 2.2.3 In‐Pack CO_2_ Measurements

The CO_2_ ratio of the gas mixture was monitored using the Witt OxyBaby (M/CO_2_) (Witten, Germany) brand in‐pack gas measurement device. The needle tip on the device adhered to the package and waited until a beep sound was heard from the device, and the results were recorded.

#### 2.2.4 Microbiological Analyses

Total mesophilic aerobic bacteria (TMAB) and total psychrophilic bacteria (TPAB) loads were determined according to AOAC (2000) (ref. no. 966.23b; 966.23c), total anaerobic bacteria TANB, *Pseudomonas* spp. (Pse), hydrogen sulfide (H_2_S)–producing bacteria (including *Shewanella putrefaciens*) (HPB), *Staphylococcus aureus* (SA), total coliform (TC), lactic acid bacteria (LAB), total yeast–mold (TYM), and *Brochothrix thermosphacta* (BrT) loads were determined according to Brown and Lowbury (1965), Halkman (2005), and Kostaki et al. (2009). Biochemical analyses (catalase, oxidase, and coagulase tests; Gram staining) were performed according to Halkman (2005).

##### 2.2.4.1 Proximate Composition

Dry matter and ash analyses were performed according to AOAC (1995) (ref. no. 925.04; 938.08), and total crude protein was determined according to the AOAC (2006) (ref. no. 954.01) Kjeldahl method using a Buchi K425 speed digester and K350 Kjeldahl distillation unit (Flawil, Switzerland). Crude fat analysis was performed according to the AOAC (2006) (ref. no. 991.36) method using a Gerhardt SE‐416 (Königswinter, Germany) fully automatic soxtherm extraction device. Carbohydrate values were calculated according to Food and Agriculture Organization (2003). Fish meat energy was calculated using the Atwater method after finding the water, fat, protein, and carbohydrate values (Sánchez‐Peña et al. 2017).

#### 2.2.5 Amino Acids and Fatty Acids Composition

Amino acid (AA) analysis after 6 N HCl digestion was carried out according to the method of high–performance liquid chromatography (HPLC) by derivatization precolumn liquid chromatography‐mass spectrometry (LC–MS/MS) using Agilent Technologies/6460 Triple Quad LC/MS (Santa Clara, CA, USA). Fatty acid composition analysis was performed according to the IID‐19 method using Thermo‐Scientific Trace 1310 Gas Chromatograph/ISQ Single Quadrupole GC–MS (Waltham, MA, USA) (International Union of Pure and Applied Chemistry 1979).

#### 2.2.6 Mineral Content of Trout Burger Patties

Mineral substances and heavy metal analysis were performed using Agilent Technologies/7700X ICP‐MS Systems (Santa Clara, CA, USA) with inductively coupled plasma mass spectrophotometer (ICP‐MS) according to the methods of Environmental Protection Agency (EPA) 200.8 and EPA 6020A (EPA 1994, 1998).

#### 2.2.7 Texture Profile Analysis and Warner–Bratzler Shear Analysis

TPA and WBS analyses were performed using Brookfield CT3 Texture Analyzer (Middleboro, MA, USA). A cylindrical steel probe (Ø = 12.20 mm) was used for TPA analysis, and the WBS Blade kit was used for WBS analysis. The settings used in the texture analyzer for TPA and WBS analyses were determined after preliminary tests and comparison with the literature. For both TPA and WBS analyses, 0.90 cm^3^ (*w* × *d* × *h*: 1 × 1 × 0.90 cm) of samples were taken from three different positions of each burger patty with a custom‐made 90° scalpel. For TPA analysis, compression rate and recovery time were set as 60% and 2 s, respectively. The trigger sensitivity was set as 0.05 N, and the probe speed was set as 2 mm/s for both TPA and WBS analyses. Regarding the load force of TPA and WBS analyses, the machine was set to 5 and 50 kg, respectively. For cooked analyses, patties were cooked for 3 min on each side in a teflon‐coated flat pan (set at 180°C), which is the standard period and temperature for the fast‐food sector.

#### 2.2.8 Scanning Electron Microscope Images

Scanning electron microscope (SEM) images (as 5000, 15,000, and 30,000 magnifications) of day‐0 were made in Selçuk University Central Laboratory (ILTEK). Imaging the days when the burger patties exceeded the consumption limits (Expiration date) was performed at Kastamonu University Central Laboratory (MERLAB). The samples were photographed under a scanning electron microscope by applying a high vacuum level.

### 2.3 Statistical Analysis

The results of the analyses were subjected to the Kolmogorov–Smirnov test, and it was determined that the data were normally distributed, and variances were found to be equal. Afterward, the data were analyzed using the one‐way analysis of variance (One‐way ANOVA). Statistical analyses were performed with a confidence interval of 95% (*p* < 0.05). Minitab 17.0 software was used for statistical evaluation of all analyses.

## 3 Results and Discussion

### 3.1 In‐Pack CO_2_ Values of Burger Patties

At the first measurement after packaging (day‐0), the amount of CO_2_ in the packaging fluctuated between 54.10% and 54.90% (*p >* 0.05) (Table 1). The study found that the CO_2_ levels in all groups decreased at different rates up to the 15th day. It can also be seen that there was a rapid decline in the control group and group B, particularly between the 6th and 9th days. The CO_2_ content decreased with fluctuations in the first 9 days of storage and began to increase after the 12th day. Randell, Hattula, and Ahvenainen (1997), Turan and Kocatepe (2013), and Kocatepe et al. (2016) also found that during the storage period, there was a significant decrease in CO_2_ levels in all groups in the first half of the storage period and a further increase from the second half onward (*p* < 0.05), similarly with our results. It can be said that these fluctuations are due to the balance between aerobic and anaerobic bacteria in the package, and this balance is maintained over time, leading to more stable conditions.

**TABLE 1:** In‐pack CO_2_ values of burger patties during storage period (%).

Days	Control	A (0.5% MTGase)	B (1% MTGase)
0	54.65 ± 0.35^Aa^	54.10 ± 0.20^Aa^	54.90 ± 0.20^Aa^
3	34.40 ± 7.40^Abc^	21.20 ± 7.10^Ab^	28.20 ± 9.98^Abc^
6	44.50 ± 0.30^Aab^	30.85 ± 0.75^Bb^	31.15 ± 0.55^Bb^
9	6.90 ± 0.80^Be^	15.60 ± 0.60^Ab^	8.00 ± 1.40^Bcd^
12	8.70 ± 1.20^Bde^	16.55 ± 1.45^Ab^	7.60 ± 0.70^Bd^
15	26.01 ± 2.20^Acd^	35.15 ± 3.95^Aab^	24.15 ± 1.15^Abcd^
18	19.05 ± 0.45^Bcde^	26.55 ± 0.45^ABb^	32.70 ± 2.50^Ab^
21	22.01 ± 5.60^Acde^	30.25 ± 6.45^Ab^	38.25 ± 1.65^Aab^
24	27.55 ± 0.55^Abc^	26.40 ± 6.30^Ab^	23.00 ± 2.31^Abcd^

*Note:* Mean (*n* = 2) ± SE. A–C (→): The difference between groups with different letters is significant (*p* < 0.05). a–e (↓): The difference between days with different letters is significant (*p* < 0.05).

### 3.2 Microbial Loads of Fish and Burger Patties

TMAB, TYM, Pse, BrT, SA, TC, TANB, HPB, LAB, and TPAB of untreated fresh fish meat were determined as 2.26, 2.61, 1.96, 1.96, < 1.00, < 1.00, < 1.00, < 1.00, < 1.00, and < 1.00 log CFU/g, respectively. The control group exceeded the TMAB limit value (6 log CFU/g) on day 18, while groups A and B exceeded the limit value on day 21 (Table 2). When evaluating TPAB loads of the burger patties, the MTGase enzyme showed a reducing effect, and due to the increasing enzyme concentration, the reducing effect was increased. In terms of Pse load, it was determined that the MTGase enzyme showed an inhibitory effect on the 0th day when the burger patties were produced (*p* < 0.05). TC bacterial load was not found (< 1 log CFU/g) in raw fish meat. As seen in Table 1, TC load increased with the addition of ingredients into the fish mince (day‐0), and it was slightly decreased and stabilized in all groups from the 6th day to the end of the storage period due to the effect of CO_2_ in the storage package. Zanganeh et al. (2021) reported that the use of essential oils inhibits the growth of microbial fauna due to both the antimicrobial activity of the essential oils used and the role of essential oils as a barrier to oxygen. *Clostridium* species are meant by anaerobes, which are of interest to food microbiology. In our research, *Clostridium* species were examined under the title of TANB. The TANB load was found to be < 1 log CFU/g from the first day (raw trout mince) of storage until the 12th day in all groups (*p* > 0.05). The TANB load of the control and A (0.5% MTGase) groups were found to be similar from the 15th day to the 24th day (*p* > 0.05), while the TANB load in group B (% 1 MTGase) remained below the lowest detectable value (< 1 log CFU/g) until the 24th day.

**TABLE 2:** Microbiological loads of uncooked burger patties during the storage period (log CFU/g).

	Groups	Day‐0	3rd day	6th day	9th day	12th day	15th day	18th day	21st day	24th day
TMAB	Control	3.04 ± 0.00^Ai^	4.13 ± 0.00^Ah^	4.46 ± 0.02^Ag^	4.77 ± 0.00^Af^	5.32 ± 0.03^Ae^	5.96 ± 0.00^Ad^	6.74 ± 0.00^Ac^	7.51 ± 0.00^Ab^	8.23 ± 0.03^Aa^
	A	3.17 ± 0.17^Ag^	4.10 ± 0.00^ABf^	3.81 ± 0.15^ABf^	4.64 ± 0.05^Ae^	5.05 ± 0.01^Ade^	5.16 ± 0.00^Bd^	5.69 ± 0.10^Bc^	6.56 ± 0.02^Bb^	8.04 ± 0.00^Aa^
	B	3.15 ± 0.01^Ag^	4.00 ± 0.04^Be^	3.62 ± 0.18^Bf^	4.19 ± 0.09^Be^	4.85 ± 0.16^Ad^	4.96 ± 0.00^Cd^	5.40 ± 0.04^Bc^	6.13 ± 0.21^Cb^	7.37 ± 0.05^Ba^
TMY	Control	3.22 ± 0.15^Ad^	4.25 ± 0.17^Ac^	3.26 ± 0.00^Ad^	3.36 ± 0.00^Ad^	4.40 ± 0.05^Ac^	4.71 ± 0.15^Ac^	5.44 ± 0.00^Ab^	5.57 ± 0.01^Ab^	6.94 ± 0.11^Aa^
	A	3.05 ± 0.05^Ae^	4.21 ± 0.05^Acd^	3.25 ± 0.06^Ae^	3.29 ± 0.03^Ae^	4.09 ± 0.09^ABd^	4.56 ± 0.00^Ac^	5.26 ± 0.00^Bb^	5.28 ± 0.14^ABb^	6.55 ± 0.13^ABa^
	B	3.15 ± 0.01^Ad^	4.02 ± 0.02^Ac^	2.94 ± 0.02^Bd^	2.61 ± 0.05^Be^	4.04 ± 0.00^Bc^	3.96 ± 0.00^Bc^	4.70 ± 0.00^Cb^	4.86 ± 0.00^Bb^	6.11 ± 0.15^Ba^
TPAB	Control	2.56 ± 0.00^Ag^	3.41 ± 0.15^Af^	4.04 ± 0.00^Ae^	4.32 ± 0.00^Ae^	4.74 ± 0.01^Ad^	6.02 ± 0.01^Ac^	6.56 ± 0.00^Ab^	7.45 ± 0.00^Aa^	7.49 ± 0.07^Aa^
	A	2.26 ± 0.00^Bi^	2.96 ± 0.00^Ah^	3.86 ± 0.00^Bg^	4.07 ± 0.00^Bf^	4.44 ± 0.00^ABe^	5.15 ± 0.01^Bd^	5.66 ± 0.00^Bc^	6.54 ± 0.02^ABb^	7.40 ± 0.03^Aa^
	B	2.35 ± 0.09^ABf^	3.11 ± 0.15^Ae^	3.91 ± 0.05^ABd^	4.00 ± 0.04^Bd^	4.26 ± 0.13^Bd^	4.55 ± 0.11^Cc^	5.07 ± 0.03^Cbc^	5.53 ± 0.27^Bb^	7.10 ± 0.06^Ba^
HPB	Control	< 1.00^Ae^	< 1.00^Ae^	2.70 ± 0.04^Ad^	3.13 ± 0.00^Ad^	3.86 ± 0.05^Ac^	4.11 ± 0.15^Abc^	4.41 ± 0.15^Ab^	4.11 ± 0.15^Abc^	5.35 ± 0.09^Aa^
	A	< 1.00^Ad^	< 1.00^Ad^	< 1.00^Bd^	2.46 ± 0.20^ABc^	3.86 ± 0.00^Ab^	3.96 ± 0.00^Ab^	3.96 ± 0.00^Ab^	3.96 ± 0.00^Ab^	5.26 ± 0.00^Aa^
	B	< 1.00^Ai^	< 1.00^Ah^	< 1.00^Bg^	2.26 ± 0.00^Bf^	3.80 ± 0.00^Ae^	3.96 ± 0.00^Ad^	3.96 ± 0.00^Ac^	3.96 ± 0.00^Ab^	4.96 ± 0.00^Ba^
Pse	Control	2.26 ± 0.00^Ae^	2.96 ± 0.00^Ad^	2.26 ± 0.00^Ae^	3.09 ± 0.09^Acd^	3.44 ± 0.00^Abc^	3.76 ± 0.20^Aab^	3.96 ± 0.04^Aa^	3.66 ± 0.00^Aab^	3.35 ± 0.09^Abcd^
	A	1.96 ± 0.00^Bd^	2.96 ± 0.00^Ab^	2.26 ± 0.00^Acd^	2.77 ± 0.03^ABbc^	3.20 ± 0.24^Aab^	3.11 ± 0.15^Aab^	3.70 ± 0.04^ABa^	3.35 ± 0.09^Aab^	3.11 ± 0.15^Aab^
	B	1.96 ± 0.00^Be^	2.96 ± 0.00^Abc^	2.26 ± 0.00^Ade^	2.56 ± 0.00^Bcd^	3.11 ± 0.15^Ab^	3.55 ± 0.11^Aa^	3.35 ± 0.09^Bab^	3.35 ± 0.09^Aab^	2.96 ± 0.00^Abc^
BrT	Control	2.55 ± 0.11^Ad^	3.71 ± 0.15^Ab^	3.07 ± 0.03^Ac^	2.66 ± 0.00^Ad^	3.86 ± 0.00^Ab^	3.80 ± 0.00^Ab^	3.96 ± 0.04^Ab^	4.89 ± 0.03^Aa^	4.91 ± 0.05^Aa^
	A	2.46 ± 0.50^Ae^	3.86 ± 0.00^Abcd^	2.96 ± 0.00^ABde^	2.26 ± 0.00^Be^	3.65 ± 0.09^Acd^	3.50 ± 0.06^Bcd^	4.02 ± 0.02^Aabc^	4.80 ± 0.00^ABab^	4.86 ± 0.05^Aa^
	B	2.87 ± 0.21^Ad^	3.46 ± 0.20^Ac^	2.86 ± 0.00^Bd^	2.26 ± 0.00^Be^	3.65 ± 0.09^Abc^	3.44 ± 0.00^Bc^	4.12 ± 0.01^Ab^	4.74 ± 0.00^Ba^	4.77 ± 0.03^Aa^
LAB	Control	< 1.00^Ad^	< 1.00^Ad^	< 1.00^Ad^	3.00 ± 0.00^Ac^	3.26 ± 0.00^Ab^	3.44 ± 0.00^Aa^	3.26 ± 0.00^Ab^	3.35 ± 0.09^Aab^	2.96 ± 0.00^Bc^
	A	< 1.00^Ac^	< 1.00^Ac^	< 1.00^Ac^	2.44 ± 0.00^Bb^	2.96 ± 0.00^Aa^	2.96 ± 0.00^Ba^	2.96 ± 0.00^Ba^	2.96 ± 0.00^Ba^	3.11 ± 0.15^Aa^
	B	< 1.00^Ac^	< 1.00^Ac^	< 1.00^Ac^	2.26 ± 0.00^Cab^	3.31 ± 0.35^Aa^	2.96 ± 0.00^Ba^	2.96 ± 0.00^Ba^	2.96 ± 0.00^Ba^	1.96 ± 0.00^Cb^
SA	Control	2.61 ± 0.05^Acd^	< 1.00^Ae^	2.41 ± 0.15^Ad^	2.56 ± 0.00^Acd^	2.86 ± 0.00^Abc^	2.56 ± 0.00^Acd^	2.70 ± 0.04^Acd^	3.16 ± 0.00^Bb^	4.26 ± 0.00^Ba^
	A	2.26 ± 0.00^Bef^	< 1.00^Ag^	< 1.00^Bg^	1.96 ± 0.00^Bf^	2.66 ± 0.00^Acd^	2.50 ± 0.06^Ade^	2.89 ± 0.15^Ac^	3.32 ± 0.00^Ab^	4.37 ± 0.00^Aa^
	B	2.26 ± 0.00^Bd^	< 1.00^Af^	< 1.00^Bf^	1.96 ± 0.00^Be^	2.35 ± 0.09^Bcd^	2.44 ± 0.00^Ac^	2.44 ± 0.00^Ac^	2.91 ± 0.00^Cb^	3.86 ± 0.00^Ca^
TC	Control	2.61 ± 0.05^Abc^	3.59 ± 0.15^Aa^	1.96 ± 0.00^Ad^	2.35 ± 0.09^Acd^	2.26 ± 0.00^Acd^	2.35 ± 0.09^Acd^	2.11 ± 0.15^Acd^	3.11 ± 0.15^Aab^	3.56 ± 0.00^Aa^
	A	2.11 ± 0.15^Ab^	2.96 ± 0.00^Ba^	1.96 ± 0.00^Ab^	2.26 ± 0.00^Ab^	1.96 ± 0.00^Bb^	2.20 ± 0.24^Ab^	2.11 ± 0.15^Ab^	2.96 ± 0.00^Aa^	3.11 ± 0.15^Aa^
	B	2.11 ± 0.15^Ab^	3.35 ± 0.09^ABa^	1.96 ± 0.00^Ab^	2.11 ± 0.15^Ab^	1.96 ± 0.00^Bb^	2.11 ± 0.15^Ab^	2.26 ± 0.00^Ab^	2.96 ± 0.00^Aa^	3.11 ± 0.15^Aa^
TANB	Control	< 1.00^Ad^	< 1.00^Ad^	< 1.00^Ad^	< 1.00^Ad^	1.96 ± 0.00^Ac^	2.96 ± 0.00^Ab^	2.96 ± 0.00^Ab^	2.96 ± 0.00^Ab^	3.26 ± 0.00^Aa^
	A	< 1.00^Ac^	< 1.00^Ac^	< 1.00^Ac^	< 1.00^Ac^	< 1.00^Bc^	2.96 ± 0.00^Ab^	2.96 ± 0.00^Ab^	2.96 ± 0.00^Ab^	3.26 ± 0.00^Aa^
	B	< 1.00^Ab^	< 1.00^Ab^	< 1.00^Ab^	< 1.00^Ab^	< 1.00^Bb^	< 1.00^Bb^	< 1.00^Bb^	< 1.00^Bb^	3.11 ± 0.15^Aa^

*Note:* Mean (*n* = 6) ± SE. A–C (↓): The difference between groups with different letters for each microorganism species is significant (*p* < 0.05). a–i (→): The difference between days with different letters for each microorganism species is significant (*p* < 0.05). A: 0.5% MTGase‐contained group, B: 1% MTGase‐containing group.

Abbreviations: BrT, *Brochothrix thermosphacta*; HPB, hydrogen sulfide (H_2_S)–producing bacteria (including *Shewanella putrefaciens*); LAB, lactic acid bacteria; Pse, *Pseudomonas* spp.; SA, *Staphylococcus aureus*; TANB, total anaerobic bacteria; TC, total coliform; TMAB, total mesophilic aerobic bacteria; TPAB, total psychrophilic bacteria; TYM, total yeast–mold.

Many researchers reported that increasing CO_2_ in the package inhibits bacterial growth (Randell, Hattula, and Ahvenainen 1997; Del Nobile et al. 2009; Kocatepe et al. 2016; Babic Milijasevic et al. 2023). It can be said that the main reason for the slower bacterial growth in our study is the MAP. Chatli et al. (2012) reported that packaging the fish with MAP effectively reduces the TPAB load in refrigerator conditions. It is possible to say that using the MTGase enzyme and MAP simultaneously showed a synergistic effect in getting a lower TPAB load in the burger patties. Various researchers reported that the MTGase enzyme has a reducing effect on the Pse—population (Tzikas et al. 2015; Aref et al. 2018). Tanavar et al. (2021) reported that coating the veal with *Mentha pulegium* and *Ocimum basilicum* essential oils prolong the shelf life compared to the control group.

One of the specific bacterial species observed in fish products that are MAP and stored in refrigerator conditions is HPB (Koutsoumanis and Nychas 1999) load. The HPB was not determined in fresh trout meat. At the end of storage, the HPB load did not exceed 5.35 log CFU/g in any group. When Table 2 is evaluated, the growth of HPB was found below < 1.00 log CFU/g in all enzyme‐contained groups in the first 6 days of storage. During storage, the lowest HPB load was determined in group B (*p* < 0.05). BrT is one of the critical spoilage bacteria, mainly observed in MAPped seafood. As shown in Table 2, slight fluctuations in BrT load were observed between groups during the storage period. Ordonez et al. (2000) reported similar findings, and they stood out in the CO_2_ tolerance of BrT. Regarding the MTGase enzyme, no studies have shown any effects on the BrT load. In our study, the inhibitory effect of the MTGase enzyme was observed since all burger patties were stored under the same MAP conditions. So, it can be said that using the MTGase enzyme was slightly effective on the BrT population. LAB are responsible for the sour taste observed during spoilage in MAPped and cold‐stored products (Nollet and Toldrá 2009). According to Özdemir and Şireli (2001), the most fundamental factor affecting LAB and BrT load may be caused by differences in production technology and personnel hygiene rather than gas combination or packaging methods. It is possible to say that the MTGase addition to the trout patty is not effective in terms of LAB load (Table 2). Generally, 10^5^–10^9^ units/g of SA in food is accepted as a “toxic level” for enterotoxin formation (Halkman 2005). To evaluate our results, particularly on days when the TMAB load exceeded 6 log CFU/g, the SA load of the groups was determined to be well below the reported toxic level. However, it can be said that the addition of MTGase enzyme has no different effect on SA load (*p* > 0.05). Sureshjani et al. (2014) reported that *Kelussia odoratissima* extract is substantially effective in inhibiting the SA. Tokay (2015) reported that the MTGase enzyme is effective in inhibiting the TC load in mackerel mince (*p* < 0.01). Although the effect of MTGase enzyme and MAP was relatively effective compared to the control group until the first half of storage, there was no positive effect in the following days. According to the results, it can be said that the use of 1% MTGase enzyme has an inhibitory effect on the TANB population. Kimura et al. (1996) reported that in terms of TANB load, the most suitable packaging method was MAP, and regarding anaerobe inhibition, they determined that the most ideal gas mixture was found as 40/60%:CO_2_/N_2_.

### 3.3 Proximate Composition of Fish and Burger Patties

While the crude lipid content of the raw fish sample was 7.12%, it was determined that the crude lipid content of the product increased with the addition of sunflower oil (4%) into the burger patties on the first day of storage (*p* < 0.05) (Table 3).

**TABLE 3:** Proximate composition (%, kcal/100 g) of raw fish and patties.

	Groups/Analyses	Crude lipid (%)	Crude protein (%)	Crude ash (%)	Moisture (%)	Carbohydrate (%)	Energy (kcal/100 g)
Day‐0	Raw sample	7.12 ± 0.00^ψ^	20.52 ± 0.02^α^	1.84 ± 0.02^Δ^	69.74 ± 0.05^α^	0.78 ± 0.06^Δ^	149.28 ± 0.15^ψ^
	Control	10.93 ± 0.06^α^	17.48 ± 0.07^θ^	2.65 ± 0.05^β^	65.44 ± 0.07^θ^	3.50 ± 0.10^α^	182.29 ± 0.13^α^
	A (0.5% MTGase)	10.45 ± 0.02^βΔ^	17.87 ± 0.10^Δ^	2.73 ± 0.06^β^	66.89 ± 0.01^β^	2.06 ± 0.03^β^	173.77 ± 0.18^βΔ^
	B (1% MTGase)	10.08 ± 0.03^θ^	18.28 ± 0.02^β^	2.75 ± 0.02^β^	66.92 ± 0.03^β^	1.96 ± 0.04^β^	171.68 ± 0.17^θ^
Expiration date	Control	10.58 ± 0.04^β^	17.59 ± 0.06^θ^	2.81 ± 0.04^αβ^	65.28 ± 0.07^θ^	3.73 ± 0.10^α^	180.52 ± 0.37^α^
	A (0.5% MTGase)	10.43 ± 0.01^Δ^	17.91 ± 0.03^Δ^	2.95 ± 0.03^α^	66.42 ± 0.23^Δ^	2.28 ± 0.25^β^	174.67 ± 1.00^β^
	B (1% MTGase)	10.07 ± 0.02^θ^	18.34 ± 0.06^β^	2.82 ± 0.03^αβ^	66.68 ± 0.02^βΔ^	2.08 ± 0.06^β^	172.32 ± 0.28^Δθ^

*Note:* Mean (*n* = 6) ± SE. α, β, Δ, θ, ψ (↓): In terms of each analysis, the difference between groups or days with different letters/symbols is significant (*p* < 0.05). Expiration date: 18 days for the control group and 21 days for groups A and B.

It is known that the main reasons for these differences are variables such as the age of the fish, the environment, and the feeding regime. Zamani et al. (2021) stated that rainbow trout's crude lipid and protein content was 7.19% and 19.45%, respectively. The crude protein amount (%) of the material used in our research (Table 3) was similar to many other studies. Cardoso et al. (2011a) found that the addition of MTGase enzyme prevented the protein loss of sea bass. Cardoso et al. (2011b) reported that the MTGase enzyme reduces protein loss and prevents protein solubilization of sea bream. The researchers concluded that the reduced protein solubility in the MTGase group was due to the cross‐linking of MHCs during setting, as it catalyzes covalent ε‐amino‐(γ‐glutamyl) lysine bonds. The ash content of the raw sample was determined to be 1.84% (Table 3). Cardoso et al. (2011a) reported that the usage of MTGase in sea bass mince had no significant effect on ash (*p* > 0.05). The energy content of all groups stored in MA conditions did not differ from day‐0 to expiration dates (18th and 21st days) (*p* > 0.05). Gaspar and Goes‐Favoni (2015) stated that MTGase modifies the meat proteins through acyl transfer reactions, which is a process that leads to the incorporation of lysine into various proteins, enhancing the nutritional value of the food.

### 3.4 Fatty Acid Compositions

It is seen (Table 4) that the burger patties that are produced with ingredients did not show any change in terms of ∑ total saturated fatty acids (∑SFA), total mono‐saturated fatty acids (∑MUFA), and total polyunsaturated fatty acids (∑PUFA) amounts both on day‐0 and on its expiration date days (*p* > 0.05). It can be said that modified atmosphere packaging is the most important reason why no significant loss in ∑SFA, ∑MUFA, and ∑PUFA contents has been observed, even at the expiration dates. Maltodextrin was present in the MTGase enzyme used in our research. Thus, maltodextrin may have contributed to the protective effect on fatty acids in addition to MTGase. More research needs to be done to verify this scenario. The amount of linoleic acid in the burger patties was increased due to the addition of sunflower oil, which is rich in linoleic acid (*p* < 0.05) (Table 4). However, neither the enzyme ratio nor the storage time significantly affected the amount of linoleic acid (*p* > 0.05). In this respect, it can be said that burger patties are very rich and nutritious products in terms of total ∑ω‐3 and ∑ω‐6 (Table 4).

**TABLE 4:** Fatty acid composition of raw material and burger patties for day‐0 and expiry dates (%).

Fatty acids (%)	Raw material	Day‐0			18th day Control	21st day A (0.5% MTGase)	21st day A (0.5% MTGase)
		Control	A (0.5% MTGase)	B (1% MTGase)			
Caproic acid (C6:0)	0.01 ± 0.00^a^	0.01 ± 0.00^a^	0.01 ± 0.00^a^	0.01 ± 0.00^a^	0.01 ± 0.00^a^	0.01 ± 0.00^a^	0.01 ± 0.00^a^
Caprylic acid (C8:0)	0.00 ± 0.00^b^	0.01 ± 0.00^a^	0.01 ± 0.00^a^	0.01 ± 0.00^a^	0.01 ± 0.00^a^	0.01 ± 0.00^a^	0.01 ± 0.00^a^
Capric acid (C10:0)	0.01 ± 0.00^a^	0.00 ± 0.00^b^	0.00 ± 0.00^b^	0.00 ± 0.00^b^	0.00 ± 0.00^b^	0.00 ± 0.00^b^	0.00 ± 0.00^b^
Lauric acid (C12:0)	0.05 ± 0.00^a^	0.04 ± 0.00^b^	0.04 ± 0.00^b^	0.04 ± 0.00^b^	0.04 ± 0.00^b^	0.04 ± 0.00^b^	0.04 ± 0.00^b^
Tridecylic acid (C13:0)	0.02 ± 0.00^a^	0.01 ± 0.00^b^	0.01 ± 0.00^b^	0.01 ± 0.00^b^	0.01 ± 0.00^b^	0.01 ± 0.00^b^	0.01 ± 0.00^b^
Myristic acid (C14:0)	2.71 ± 0.11^a^	2.04 ± 0.02^b^	1.99 ± 0.02^b^	1.94 ± 0.02^b^	2.05 ± 0.02^b^	1.95 ± 0.03^b^	1.91 ± 0.02^b^
Pentadecylic acid (C15:0)	0.46 ± 0.03^a^	0.30 ± 0.01^b^	0.30 ± 0.00^b^	0.29 ± 0.01^b^	0.30 ± 0.01^b^	0.29 ± 0.01^b^	0.29 ± 0.01^b^
Palmitic acid (C16:0)	11.01 ± 0.21^a^	10.14 ± 0.06^bc^	10.02 ± 0.09^bc^	9.87 ± 0.05^bc^	10.17 ± 0.05^b^	9.99 ± 0.07b^c^	9.77 ± 0.12^c^
Margaric acid (C17:0)	0.64 ± 0.01^a^	0.52 ± 0.01^b^	0.50 ± 0.01^b^	0.50 ± 0.01^b^	0.51 ± 0.01^b^	0.49 ± 0.01^b^	0.50 ± 0.00^b^
Stearic acid (C18:0)	8.03 ± 0.20^a^	8.20 ± 0.11^a^	8.20 ± 0.08^a^	8.16 ± 0.06^a^	8.10 ± 0.15^a^	8.07 ± 0.18^a^	8.09 ± 0.08^a^
Arachidic acid (C20:0)	0.80 ± 0.02^b^	0.97 ± 0.02^a^	0.97 ± 0.01^a^	0.98 ± 0.01^a^	1.02 ± 0.04^a^	1.01 ± 0.02^a^	1.00 ± 0.01^a^
Heneicosilicic acid (C21:0)	0.02 ± 0.02^a^	0.03 ± 0.00^a^	0.04 ± 0.00^a^	0.03 ± 0.00^a^	0.04 ± 0.00^a^	0.03 ± 0.00^a^	0.03 ± 0.00^a^
Behenic acid (C22:0)	0.38 ± 0.01^c^	1.15 ± 0.01^b^	1.22 ± 0.02^a^	1.27 ± 0.01^a^	1.14 ± 0.02^b^	1.27 ± 0.02^a^	1.26 ± 0.01^a^
Trichosilic acid (C23:0)	0.05 ± 0.00^a^	0.03 ± 0.00^b^	0.03 ± 0.00^b^	0.03 ± 0.00^b^	0.03 ± 0.00^b^	0.03 ± 0.00^b^	0.02 ± 0.00^b^
Lignoceric acid (C24:0)	0.70 ± 0.03^ab^	0.64 ± 0.05^b^	0.90 ± 0.01^a^	0.90 ± 0.01^a^	0.62 ± 0.05^b^	0.79 ± 0.08^ab^	0.75 ± 0.08^ab^
∑SFA (saturated fatty acids)	24.90 ± 0.61^a^	24.09 ± 0.21^a^	24.24 ± 0.22^a^	24.04 ± 0.12^a^	24.03 ± 0.25^a^	23.98 ± 0.38^a^	23.68 ± 0.22^a^
Myristoleic acid (C14:1)	0.17 ± 0.01^a^	0.11 ± 0.00^b^	0.11 ± 0.00^b^	0.10 ± 0.00^b^	0.11 ± 0.00^b^	0.10 ± 0.00^b^	0.10 ± 0.00^b^
Pentadeconoic acid (C15:1)	0.12 ± 0.04^a^	0.04 ± 0.00^b^	0.04 ± 0.00^b^	0.04 ± 0.00^b^	0.04 ± 0.00^b^	0.04 ± 0.00^b^	0.04 ± 0.00^b^
Palmitoleic acid (C16:1)	0.43 ± 0.01^a^	0.33 ± 0.01^b^	0.32 ± 0.00^b^	0.31 ± 0.01^b^	0.33 ± 0.01^b^	0.31 ± 0.02^b^	0.31 ± 0.01^b^
Heptadeconoic acid (C17:1)	0.55 ± 0.01^a^	0.43 ± 0.01^c^	0.51 ± 0.01^ab^	0.47 ± 0.02^bc^	0.44 ± 0.01^c^	0.44 ± 0.02^c^	0.51 ± 0.01^ab^
Oleic acid (C18:1n9c)	19.04 ± 0.12^b^	21.86 ± 0.59^ab^	22.53 ± 0.53^a^	22.06 ± 0.50^ab^	21.26 ± 0.45^ab^	22.04 ± 0.78^ab^	22.43 ± 0.56^a^
Elaidic acid (C18:1n9t)	3.55 ± 0.14^a^	3.11 ± 0.11^a^	3.06 ± 0.07^a^	2.99 ± 0.07^a^	3.31 ± 0.16^a^	3.19 ± 0.15^a^	2.99 ± 0.16^a^
Erucic acid (C22:1n9)	2.73 ± 0.06^a^	2.13 ± 0.02^b^	2.11 ± 0.02^b^	2.09 ± 0.01^b^	2.13 ± 0.02^b^	2.13 ± 0.03^b^	2.10 ± 0.02^b^
Nervonic acid (C24:1)	0.92 ± 0.03^a^	0.69 ± 0.01^b^	0.71 ± 0.02^b^	0.72 ± 0.02^b^	0.71 ± 0.03^b^	0.69 ± 0.03^b^	0.71 ± 0.03^b^
∑MUFA (monounsaturated fatty acids)	31.78 ± 0.14^a^	32.47 ± 0.49^a^	33.07 ± 0.42^a^	32.42 ± 0.41^a^	32.16 ± 0.34^a^	32.70 ± 0.70^a^	32.86 ± 0.38^a^
Linolelaidic acid (C18:2n6t)	0.56 ± 0.00^a^	0.51 ± 0.00^b^	0.46 ± 0.01^d^	0.46 ± 0.00^d^	0.50 ± 0.01^bc^	0.47 ± 0.01^cd^	0.47 ± 0.01^d^
Linoleic acid (C18:2n6c)	14.31 ± 0.07^b^	19.13 ± 0.26^a^	18.83 ± 0.41^a^	19.79 ± 0.25^a^	19.60 ± 0.33^a^	19.62 ± 1.03^a^	20.27 ± 0.38^a^
α‐Linolenic acid (C18:3n3)	5.36 ± 0.05^a^	4.32 ± 0.03^b^	4.25 ± 0.05^b^	4.20 ± 0.04^b^	4.36 ± 0.03^b^	4.30 ± 0.03^b^	4.19 ± 0.08^b^
γ‐Linolenic acid (C18:3n6)	0.92 ± 0.01^a^	0.59 ± 0.01^b^	0.56 ± 0.01^b^	0.56 ± 0.00^b^	0.58 ± 0.01^b^	0.58 ± 0.02^b^	0.59 ± 0.01^b^
cis‐11‐Eicosenioic acid (C20:1)	4.28 ± 0.06^a^	3.77 ± 0.03^bc^	3.70 ± 0.04^bc^	3.65 ± 0.03^c^	3.85 ± 0.03^b^	3.77 ± 0.02^bc^	3.68 ± 0.06^bc^
cis‐11,14‐Eicosadienoic acid (C20:2)	3.21 ± 0.03^a^	2.84 ± 0.02^b^	2.82 ± 0.03^b^	2.78 ± 0.02^b^	2.87 ± 0.02^b^	2.82 ± 0.02^b^	2.78 ± 0.03^b^
cis‐11,14,17‐Eicosatrienoic acid (C20:3n3)	0.92 ± 0.01^a^	0.78 ± 0.01^b^	0.75 ± 0.01^b^	0.75 ± 0.00^b^	0.83 ± 0.05^ab^	0.75 ± 0.02^b^	0.74 ± 0.01^b^
Dihomogamalinoleic acid (C20:3n6)	1.88 ± 0.01^a^	1.46 ± 0.02^b^	1.46 ± 0.02^b^	1.46 ± 0.01^b^	1.44 ± 0.03^b^	1.44 ± 0.03^b^	1.44 ± 0.01^b^
Arachidonic acid (C20:4n6)	2.14 ± 0.03^a^	1.71 ± 0.02^b^	1.70 ± 0.02^b^	1.69 ± 0.01^b^	1.68 ± 0.02^b^	1.68 ± 0.02^b^	1.63 ± 0.02^b^
Eicosapentaenoic acid (C20:5n3)	4.68 ± 0.05^a^	4.05 ± 0.04^b^	3.88 ± 0.04^bc^	3.87 ± 0.03^bc^	3.96 ± 0.03^b^	3.77 ± 0.04^c^	3.70 ± 0.07^c^
cis‐13,16‐Docosadienoic acid (C22:2)	0.45 ± 0.01^a^	0.34 ± 0.01^b^	0.34 ± 0.00^b^	0.33 ± 0.00^b^	0.33 ± 0.01^b^	0.34 ± 0.01^b^	0.34 ± 0.01^b^
Docosahexaenoic acid (C22:6n3)	8.88 ± 0.24^a^	7.72 ± 0.12^b^	7.66 ± 0.16^b^	7.65 ± 0.10^b^	7.72 ± 0.17^b^	7.60 ± 0.14^b^	7.31 ± 0.18^b^
∑PUFA (polyunsaturated fatty acids)	43.30 ± 0.47^a^	43.44 ± 0.46^a^	42.69 ± 0.40^a^	43.53 ± 0.38^a^	43.87 ± 0.53^a^	43.36 ± 1.04^a^	43.45 ± 0.42^a^
Identified	99.98 ± 0.01^a^	99.99 ± 0.01^a^	99.99 ± 0.01^a^	99.99 ± 0.01^a^	100.06 ± 0.05^a^	100.03 ± 0.05^a^	99.99 ± 0.00^a^
∑ω3	19.84 ± 0.33^a^	16.87 ± 0.18^b^	16.53 ± 0.25^b^	16.47 ± 0.15^b^	16.87 ± 0.26^b^	16.41 ± 0.17^b^	15.94 ± 0.31^b^
∑ω6	19.80 ± 0.10^b^	23.40 ± 0.27^a^	23.00 ± 0.38^a^	23.95 ± 0.24^a^	23.80 ± 0.28^a^	23.79 ± 0.98^a^	24.40 ± 0.34^a^
ω6/ω3 ratio	1.00 ± 0.01^c^	1.39 ± 0.00^b^	1.39 ± 0.03^b^	1.45 ± 0.01^ab^	1.41 ± 0.01^ab^	1.45 ± 0.06^ab^	1.53 ± 0.04^a^

*Note:* Mean (*n* = 6) ± SE. a–g (→): The difference between groups and days with different letters is significant (*p* < 0.05).

It was determined that an increase in the storage time and an increase in the MTGase amount led to an increase in the omega‐3 (∑ω‐3) and total omega‐6 (∑ω‐6) ratio (Table 4). According to Rasaei et al. (2023), who performed genotypic studies in women, the incidence of health problems such as depression, anxiety, stress, and inflammation increased significantly with increasing ω‐6 intake. The researchers also stated that an increase in ω‐3 intake can significantly prevent these diseases and eliminate many related cardiovascular and brain health risks. It is suggested that the optimal ω‐6 to ω‐3 ratio should be between 1:1 and 5:1 to ensure a healthy balance (Gonzalez‐Becerra et al. 2023). Despite the addition of the ingredients into the burger patties, the fact that the ω‐6 to ω‐3 ratio is still found between 1.39 and 1.53 even on its expiration dates is a strong indication that the product is already a precious food in terms of heart and brain health.

### 3.5 Amino Acid Compositions

When the AA results are evaluated together, the total protein ratio in burger patties decreases (lower‐level decreases were seen in the groups with enzymes) with adding ingredients to fresh fish mince. However, it was determined that changes in the amount of lysine (except for the control group), histidine, and threonine occurred in a minimal amount, but a decrease was observed in the content of all other essential AAs (Table 5).

**TABLE 5:** The results of amino acids and their derivatives of raw material and burger patties for day‐0 and expiry dates (g/100 g).

	Raw material	Day‐0			18th day Control	21st day A (0.5% MTGase)	21st day B (1% MTGase)
		Control	A (0.5% MTGase)	B (1% MTGase)			
Essential amino acids							
Histidine	0.95 ± 0.05^a^	0.81 ± 0.03^ab^	0.69 ± 0.05^b^	0.75 ± 0.04^ab^	0.79 ± 0.04^ab^	0.64 ± 0.07^b^	0.76 ± 0.04^ab^
Isoleucine	1.06 ± 0.02^a^	0.72 ± 0.01^b^	0.69 ± 0.02^b^	0.69 ± 0.01^b^	0.73 ± 0.08^b^	0.73 ± 0.23^b^	0.71 ± 0.04^b^
Leucine	2.02 ± 0.06^a^	1.50 ± 0.05^b^	1.53 ± 0.18^b^	1.32 ± 0.01^b^	1.52 ± 0.03^b^	1.39 ± 0.08^b^	1.47 ± 0.08^b^
Lysine	3.38 ± 0.13^a^	2.41 ± 0.17^b^	2.92 ± 0.17^ab^	2.80 ± 0.08^ab^	2.49 ± 0.03^b^	2.81 ± 0.38^ab^	2.83 ± 0.07^ab^
Methionine	0.87 ± 0.02^a^	0.65 ± 0.03^b^	0.63 ± 0.02^bc^	0.59 ± 0.01^bc^	0.66 ± 0.01^b^	0.54 ± 0.10^c^	0.63 ± 0.02^bc^
Arginine	1.57 ± 0.02^a^	1.24 ± 0.04^b^	1.22 ± 0.06^bc^	1.24 ± 0.02^b^	1.22 ± 0.05^bc^	1.13 ± 0.34^bc^	1.10 ± 0.04^c^
Phenylalanine	1.29 ± 0.01^a^	1.03 ± 0.06^b^	0.98 ± 0.04^b^	0.94 ± 0.02^b^	1.06 ± 0.05^b^	0.90 ± 0.17^b^	0.96 ± 0.03^b^
Threonine	1.46 ± 0.14^a^	1.17 ± 0.02^ab^	1.07 ± 0.04^ab^	0.97 ± 0.03^b^	1.08 ± 0.01^ab^	0.86 ± 0.23^b^	1.00 ± 0.02^ab^
Valine	1.53 ± 0.04^a^	1.19 ± 0.05^b^	1.04 ± 0.03^bc^	1.01 ± 0.06^c^	1.18 ± 0.14^bc^	1.11 ± 0.13^bc^	1.12 ± 0.06^bc^
∑EAA	14.13 ± 0.15^a^	10.72 ± 0.33^b^	10.77 ± 0.24^b^	10.31 ± 0.34^b^	10.73 ± 0.31^b^	10.11 ± 1.79^b^	10.58 ± 0.53^b^
Non‐essential amino acids							
Aspartic acid	2.28 ± 0.03^a^	1.53 ± 0.12^b^	1.65 ± 0.17^ab^	1.63 ± 0.11^ab^	1.80 ± 0.14^ab^	1.67 ± 0.49^ab^	1.60 ± 0.05^ab^
Glutamic acid	3.72 ± 0.00^a^	2.83 ± 0.05^b^	3.08 ± 0.12^ab^	3.19 ± 0.03^ab^	2.90 ± 0.05^b^	3.05 ± 0.18^ab^	3.11 ± 0.14^ab^
Serine	1.21 ± 0.05^a^	0.99 ± 0.02^b^	0.96 ± 0.05^b^	0.97 ± 0.06^b^	0.98 ± 0.03^b^	0.83 ± 0.23^b^	0.97 ± 0.00^b^
Proline	1.02 ± 0.01^a^	0.87 ± 0.04^b^	0.89 ± 0.08^b^	0.89 ± 0.15^b^	0.87 ± 0.01^b^	0.89 ± 0.04^b^	0.84 ± 0.08^b^
Glycine	1.30 ± 0.28^a^	1.08 ± 0.03^a^	1.10 ± 0.17^a^	1.20 ± 0.19^a^	1.20 ± 0.17^a^	1.06 ± 0.39^a^	1.18 ± 0.07^a^
Alanine	1.74 ± 0.01^a^	1.26 ± 0.02^b^	1.21 ± 0.11^bc^	1.25 ± 0.01^b^	1.32 ± 0.10^b^	1.15 ± 0.48^c^	1.21 ± 0.04^bc^
Tyrosine	0.83 ± 0.03^a^	0.58 ± 0.01^b^	0.62 ± 0.03^ab^	0.60 ± 0.07^ab^	0.65 ± 0.04^ab^	0.61 ± 0.27^ab^	0.60 ± 0.01^ab^
Cysteine	0.26 ± 0.03^a^	0.15 ± 0.01^b^	0.23 ± 0.02^a^	0.18 ± 0.01^ab^	0.19 ± 0.01^ab^	0.17 ± 0.03^ab^	0.24 ± 0.00^a^
∑NEAA	12.36 ± 0.38^a^	9.29 ± 0.22^b^	9.74 ± 0.31^b^	9.91 ± 0.27^b^	9.90 ± 0.26^b^	9.43 ± 1.08^b^	9.75 ± 0.19^b^
EAA/NEAA	1.14	1.15	1.11	1.04	1.08	1.07	1.09
Others							
1‐Metilhistidine	0.48 ± 0.01^a^	0.28 ± 0.01^cd^	0.29 ± 0.03^bc^	0.28 ± 0.02^cd^	0.33 ± 0.01^b^	0.28 ± 0.07^cd^	0.26 ± 0.03^d^
3‐Aminoisobutyric acid	0.04 ± 0.00^a^	0.04 ± 0.00^a^	0.04 ± 0.00^a^	0.04 ± 0.00^a^	0.04 ± 0.00^a^	0.04 ± 0.02^a^	0.04 ± 0.00^a^
3‐Methylhistidine	0.13 ± 0.00^a^	0.13 ± 0.00^a^	0.12 ± 0.00^a^	0.12 ± 0.00^a^	0.13 ± 0.00^a^	0.05 ± 0.00^b^	0.13 ± 0.00^a^
β‐Alanine	0.22 ± 0.01^a^	0.15 ± 0.00^b^	0.14 ± 0.02^b^	0.14 ± 0.01^b^	0.14 ± 0.02^b^	0.13 ± 0.05^b^	0.13 ± 0.01^b^
Homocysteine	0.02 ± 0.00^a^	0.02 ± 0.00^a^	0.02 ± 0.00^a^	0.02 ± 0.00^a^	0.02 ± 0.00^a^	0.01 ± 0.00^b^	0.02 ± 0.00^a^
Ethanolamine	0.03 ± 0.00^a^	0.02 ± 0.00^ab^	0.02 ± 0.00^ab^	0.02 ± 0.00^ab^	0.02 ± 0.00^ab^	0.01 ± 0.00^b^	0.02 ± 0.00^ab^
2‐Aminobutyric acid	0.03 ± 0.00^a^	0.03 ± 0.00^a^	0.03 ± 0.00^a^	0.03 ± 0.00^a^	0.03 ± 0.00^a^	0.02 ± 0.01^a^	0.03 ± 0.00^a^
Anserine	0.02 ± 0.00^a^	0.02 ± 0.00^a^	0.02 ± 0.00^a^	0.02 ± 0.00^a^	0.02 ± 0.00^a^	0.02 ± 0.01^a^	0.02 ± 0.00^a^
Carnosine	0.22 ± 0.00^a^	0.22 ± 0.00^a^	0.22 ± 0.00^a^	0.22 ± 0.00^a^	0.22 ± 0.00^a^	0.08 ± 0.00^b^	0.22 ± 0.00^a^
Norvaline	0.04 ± 0.00^a^	0.03 ± 0.00^a^	0.03 ± 0.00^a^	0.03 ± 0.00^a^	0.04 ± 0.00^a^	0.04 ± 0.01^a^	0.03 ± 0.00^a^
Ornithine	0.13 ± 0.00^b^	0.13 ± 0.00^ab^	0.13 ± 0.00^ab^	0.14 ± 0.00^ab^	0.14 ± 0.01^ab^	0.05 ± 0.00^c^	0.14 ± 0.00^a^
trans‐4‐Hydroxyproline	0.12 ± 0.00^b^	0.10 ± 0.00^c^	0.12 ± 0.01^b^	0.26 ± 0.07^a^	0.09 ± 0.01^c^	0.08 ± 0.01^d^	0.12 ± 0.00^b^

*Note:* Mean (*n* = 6) ± SE. a–g (→): The difference between groups and days with different letters is significant (*p* < 0.05).

According to De Jong and Koppelman (2002), the TGase enzyme increases the nutritional value of food by catalyzing the acyl‐transfer reaction, which leads to inter‐ or intramolecular cross‐linking and ensures that the ɛ‐amino group of lysine in proteins are incorporated into the food. This can be explained by enzyme utilization's limited effect on increasing or decreasing the proportional amount of essential amino acids. Fang et al. (2019) reported that lysine and glutamic acid levels were slightly higher in silver carp surimi in the enzyme‐used groups than in the control group. The AA found at the maximum level in all enzyme‐contained groups was glutamic acid (Table 5). The lowest glutamic acid amount was found in the control group. The storage period did not cause any change in the glutamic acid amount (*p* > 0.05). On the other hand, it is seen that increasing the enzyme concentration and/or storage period was not effective in terms of glutamic acid amount in enzyme‐contained groups (*p* > 0.05). Glutamate is the free AA form of glutamic acid. It is associated with the fifth taste called umami (Chaudhari, Pereira, and Roper 2009). It can be said that using MTGase increased the umami effect, but using the higher concentration did not provide any significant benefit. The ∑EAA/∑NEAA ratio was highest in the Control (1.15) on day‐0, closest to the ratio of raw fish determined as 1.14 (Table 5). Some AAs (lysine, glutamic acid, tyrosine, etc.) were determined to be slightly higher in enzyme‐contained groups than in the control group. The estimated daily intakes of lysine, threonine, and methionine are 75.09, 43.14, and 25.29 mg/kg of body weight per day, respectively (Blachier et al. 2021). Upon comparing the averages of these AA amounts of the MTGase‐containing groups, a person with a body weight of 70 kg will meet approximately 82% of the lysine requirement, 50% of the threonine requirement, and 78% of the methionine requirement from 150 g of burger patties.

### 3.6 Mineral Contents

While the transglutaminase enzyme is isolated from mammals or fish, its ability to carry out activation and cross‐linking reactions depends entirely on the Ca^+2^ concentration in its environment. It is a big problem when the amount of Ca^+2^ in the environment is low or when Ca^+2^ ions are necessary for product quality (Ashie and Lanier 2000). However, MTGase (by Ajinomoto Corp.) enzyme, which is obtained from various bacteria species such as *S. mobaraense*, *C. glutamicum* or its strains by DNA modification, which works entirely independently of the presence of Ca^+2^ (Ashie and Lanier 2000). Although the functional principle of the MTGase enzyme is completely independent of Ca^+2^ ions, a decrease in Ca^+2^ can be observed during proteolytic degradation from plasma, albeit in very small amounts. However, this decline is generally insignificant (Ashie and Lanier 2000; Gaspar and Goes‐Favoni 2015). While MTGase enzyme activity increases in the presence of Co^+3^, Ba^+2^, and K+, cysteine in the active site is inhibited by Zn^+2^, Cu^+2^, Hg^+^, and Pb^+2^ ions bound to its thiol group (Motoki and Seguro 1998). Compared to the control, Cu^+2^ and Zn^+2^ levels were found to be lower in both enzyme‐containing groups on day‐0 and expiry days (*p* < 0.05) (Table 6). In general, the mineral content of all groups was increased on the expiration days compared to the results on day‐0, and it can be said that this situation is related to water loss. Salt is a water‐binding agent that positively affects food yield due to its water‐binding properties (Pietrasik and Li‐Chan 2002). Salt is used as a water‐binding agent in fish meats with high water content, but the content of salt/Na^+2^ in the final product is high. The burger patties prepared in the research (150 g) met the daily intake requirement of Mg^+2^, Ca^+2^, K^+^, and Fe^+2^ at approximately 13%, 4%, 12%, and 15%, respectively (Godswill et al. 2020). Hg^+^, Cd^+2^, and Pb^+2^ amounts were found below detectable values (< 0.01 mg/kg) of raw trout mince and all groups on both production and expiration days (*p* > 0.05).

**TABLE 6:** Mineral composition of raw material and burger patties (mg/kg).

Minerals (mg/kg)	Raw material	Day‐0			18th day Control	21st day A (0.5% MTGase)	21st day B (1% MTGase)
		Control	A (0.5% MTGase)	B (1% MTGase)			
Sodium (Na)	246.59 ± 4.23^g^	5641.03 ± 73.33^f^	6683.50 ± 127.76^d^	8082.92 ± 47.31^b^	6298.09 ± 125.95^e^	7179.94 ± 118.02^c^	8457.61 ± 142.28^a^
Magnesium (Mg)	245.82 ± 3.70^d^	320.31 ± 3.78^c^	333.60 ± 2.70^bc^	340.90 ± 3.31^b^	379.48 ± 4.66^a^	345.69 ± 4.81^b^	345.84 ± 1.99^b^
Potassium (K)	3412.82 ± 46.75^d^	3492.96 ± 20.61^d^	3597.64 ± 46.24^cd^	3691.12 ± 28.94^bc^	3886.63 ± 44.08^a^	3724.08 ± 49.02^ab^	3822.38 ± 49.83^ab^
Calcium (Ca)	61.92 ± 2.43^c^	394.34 ± 86.11^a^	354.76 ± 81.46^a^	233.64 ± 15.90^b^	485.05 ± 109.57^a^	184.27 ± 8.87^b^	207.97 ± 2.34^b^
Iron (Fe)	5.75 ± 0.04^d^	12.91 ± 1.27^c^	19.45 ± 1.61^b^	21.66 ± 2.62^a^	28.53 ± 3.92^a^	12.19 ± 0.11^c^	12.97 ± 0.88^c^
Rubidium (Rb)	3.44 ± 0.03^a^	2.68 ± 0.02^b^	2.69 ± 0.03^b^	2.70 ± 0.01^b^	2.88 ± 0.06^b^	2.37 ± 0.16^c^	2.68 ± 0.03^b^
Lithium (Li)	0.02 ± 0.01^c^	0.09 ± 0.01^b^	0.11 ± 0.01^ab^	0.12 ± 0.01^ab^	0.15 ± 0.01^a^	0.12 ± 0.01^a^	0.13 ± 0.01^ab^
Beryllium (Be)	0.02 ± 0.00^c^	0.03 ± 0.00^bc^	0.03 ± 0.00^a^	0.03 ± 0.00^abc^	0.03 ± 0.00^ab^	0.03 ± 0.00^ab^	0.03 ± 0.00^ab^
Vanadium (V)	0.02 ± 0.00^b^	0.03 ± 0.00^ab^	0.04 ± 0.00^a^	0.03 ± 0.00^ab^	0.04 ± 0.01^a^	0.03 ± 0.00^ab^	0.03 ± 0.00^ab^
Chromium (Cr)	0.04 ± 0.00^b^	0.08 ± 0.01^ab^	0.13 ± 0.02^ab^	0.12 ± 0.01^ab^	0.16 ± 0.04^a^	0.06 ± 0.00^b^	0.08 ± 0.00^ab^
Manganese (Mn)	0.14 ± 0.01^c^	1.40 ± 0.05^ab^	1.35 ± 0.06^b^	1.52 ± 0.02^ab^	1.90 ± 0.27^a^	1.02 ± 0.01^b^	1.04 ± 0.05^b^
Nickel (Ni)	0.15 ± 0.00^bc^	0.21 ± 0.03^ab^	0.17 ± 0.01^bc^	0.26 ± 0.01^a^	0.16 ± 0.02^bc^	0.13 ± 0.01^c^	0.17 ± 0.00^bc^
Gallium (Ga)	0.02 ± 0.00^a^	0.02 ± 0.00^a^	0.02 ± 0.00^a^	0.02 ± 0.00^a^	0.02 ± 0.00^a^	0.02 ± 0.00^a^	0.02 ± 0.00^a^
Selenium (Se)	0.19 ± 0.00^a^	0.20 ± 0.01^a^	0.19 ± 0.01^a^	0.21 ± 0.01^a^	0.22 ± 0.01^a^	0.19 ± 0.02^a^	0.22 ± 0.01^a^
Strontium (Sr)	0.13 ± 0.00^c^	1.28 ± 0.20^ab^	1.20 ± 0.19^ab^	0.89 ± 0.01^abc^	1.49 ± 0.26^a^	0.69 ± 0.04^bc^	0.84 ± 0.01^abc^
Cesium (Cs)	0.05 ± 0.00^a^	0.04 ± 0.00^bc^	0.05 ± 0.00^bc^	0.05 ± 0.00^bc^	0.05 ± 0.00^b^	0.04 ± 0.00^c^	0.05 ± 0.00^bc^
Barium (Ba)	0.07 ± 0.00^c^	0.27 ± 0.01^b^	0.27 ± 0.01^b^	0.31 ± 0.02^ab^	0.40 ± 0.06^a^	0.20 ± 0.02^bc^	0.27 ± 0.00^b^
Thallium (Tl)	0.02 ± 0.00^a^	0.02 ± 0.00^b^	0.01 ± 0.00^c^	0.01 ± 0.00^d^	0.01 ± 0.00^d^	0.01 ± 0.00^d^	0.01 ± 0.00^d^
Zinc (Zn)	3.24 ± 0.06^c^	4.81 ± 0.07^a^	4.12 ± 0.12^b^	4.16 ± 0.09^bc^	5.15 ± 0.37^a^	3.72 ± 0.08^c^	4.15 ± 0.07^bc^
Aluminum (Al)	1.20 ± 0.04^b^	8.85 ± 0.52^a^	12.37 ± 1.93^a^	9.14 ± 0.29^a^	11.47 ± 1.20^a^	9.25 ± 0.10^a^	9.15 ± 0.47^a^
Copper (Cu)	0.42 ± 0.00^c^	0.94 ± 0.13^a^	0.70 ± 0.03^bc^	0.66 ± 0.01^bc^	0.73 ± 0.03^a^	0.62 ± 0.01^bc^	0.62 ± 0.01^bc^
Arsenic (As)	0.41 ± 0.00^a^	0.36 ± 0.01^b^	0.34 ± 0.01^b^	0.34 ± 0.01^b^	0.42 ± 0.02^a^	0.34 ± 0.01^b^	0.34 ± 0.01^b^
Cobalt (Co)	0.02 ± 0.00^c^	0.03 ± 0.00^bc^	0.03 ± 0.00^bc^	0.03 ± 0.00^bc^	0.04 ± 0.00^a^	0.03 ± 0.00^bc^	0.03 ± 0.00^b^
Lead (Pb)	< 0.01	< 0.01	< 0.01	< 0.01	< 0.01	< 0.01	< 0.01
Cadmium (Cd)	< 0.01	< 0.01	< 0.01	< 0.01	< 0.01	< 0.01	< 0.01
Mercury (Hg)	< 0.01	< 0.01	< 0.01	< 0.01	< 0.01	< 0.01	< 0.01

*Note:* Mean (*n* = 6) ± SE. a–g (↔): The difference between groups and days with different letters is significant (*p* < 0.05).

### 3.7 Texture Profile Analysis and Warner–Bratzler Shear Changes Burger Patties

Moreno, Borderías, and Baron (2010) reported that using the MTGase enzyme in the fishery product increases the hardness value and storage time. Various researchers also reported that the MTGase enzyme is increasing the hardness values (Moreno, Carballo, and Borderias 2008; Andres‐Bello et al. 2011; Kunnath et al. 2015; Gore et al. 2023). However, Tzikas et al. (2015) noted that MTGase enzyme activation is dependent on salt content in fish meat, and using the MTGase enzyme without salt or using salt under the ratio of 1% results in insufficient formation of cross‐links and important mechanical properties such as hardness and springiness. It can be said that this result is related to the fact that the MTGase enzyme reduces surface stickiness. Moreno, Carballo, and Borderias (2008) and Andres‐Bello et al. (2011) reported that using MTGase in fish mince is decreasing a considerable ratio of adhesiveness. Using the MTGase increases springiness, and increasing the enzyme concentration provides more elastic restructured fishery products (Moreno, Carballo, and Borderias 2008; Andres‐Bello et al. 2011; Kunnath et al. 2015; Tzikas et al. 2015). Regarding the comparison of cohesiveness results between the 0th and 24th days, the values of group B remained similar (*p* > 0.05), while control and group A decreased compared to the last day of storage (*p* < 0.05) (Table 7). Various researchers (Andres‐Bello et al. 2011; Kunnath et al. 2015; Tzikas et al. 2015) reported that using the MTGase enzyme increases cohesiveness values, and increasing the enzyme concentration causes higher cohesiveness values. Kunnath et al. (2015) reported that the MTGase enzyme increases chewiness (*p* < 0.05). Additionally, Andres‐Bello et al. (2011) noted that enhancing the enzyme concentration increases chewiness as well (*p* < 0.05). Using the MTGase enzyme increases the hardness value of cooked/high‐temperature applied restructured fishery products (Tzikas et al. 2015). One of the main reasons for this is the partial or complete loss of negative penetration stress in the restructured product formation that contains the MTGase enzyme. Another factor, as reported by Del Pulgar, Gázquez, and Ruiz‐Carrascal (2012), is that the humidity of the product decreases due to the temperature in the cooking process, denaturation begins to occur, and accordingly, the “negative adhesive area” between the texture machine probe and the burger patties decreases to very low levels or disappears completely. When studies conducted by many researchers on texture were examined, it was seen that adhesiveness was either detected very low or could not be detected at all in heat‐treated fishery products, especially when texture‐improving substances (enzyme, salt, sodium caseinate, etc.) were used. Various researchers (Tzikas et al. 2015; Fang et al. 2019) reported that the MTGase enzyme is slightly effective in the resilience parameter in restructured fishery products. It can be said that the effect of MTGase is very limited on the resilience parameter in heat‐treated fishery products. At the end of the storage period, a decrease was observed in all groups (Table 7) compared to the initial values, with the slightest decrease in group B (*p* < 0.05). The increasing effect and the concentration difference of the enzyme on springiness are clearly seen (*p* < 0.05). Researchers (Tellez‐Luis et al. 2002; Tzikas et al. 2015) reported that MTGase has an increasing effect on the springiness parameter in restructured fishery products. Tellez‐Luis et al. (2002) determined higher cohesiveness results in the group that used the enzyme at a low concentration (0.3%) than the high‐concentration (0.6%) group in restructured products from silver carp. During the storage period, the highest and lowest chewiness (N × mm) results were found in group B and the control group, respectively (*p* < 0.05) (Table 7). To compare the chewiness values of the final storage day (24th day) with initial values (day‐0), a decrease was observed in the groups of control and B, while group A remained unchanged (*p* < 0.05). Çiftçi (2010) reported that the TGase enzyme increases the chewiness parameter in cooked fish balls, and increasing the enzyme concentration results in higher chewiness results (*p* < 0.05). Since there is some fish gelatin in the enzyme product we used, it may have affected some TPA parameters.

**TABLE 7:** TPA and WBS results of uncooked and cooked burger patties.

Groups/Days			Day‐0	3rd day	6th day	9th day	12th day	15th day	18th day	21st day	24th day
Hardness (N)	Control	Uncooked	2.03 ± 0.07^C^_cd_	2.45 ± 0.10^B^_a_	2.39 ± 0.03^C^_ab_	2.42 ± 0.09^C^_ab_	2.12 ± 0.03^C^_bc_	1.73 ± 0.07^C^_de_	1.44 ± 0.07^C^_e_	1.60 ± 0.07^C^_e_	1.60 ± 0.03^C^_e_
		Cooked	25.07 ± 0.56^Z^_1_	24.25 ± 0.57^Z^_1_	23.18 ± 0.43^Z^_1_	21.15 ± 0.12^Z^_2_	19.19 ± 0.35^Z^_3_	15.36 ± 0.28^Z^_4_	13.14 ± 0.32^Z^_5_	14.45 ± 0.24^Z^_4,5_	8.66 ± 0.40^Z^_6_
	A	Uncooked	4.31 ± 0.10^B^_d_	6.41 ± 0.09^A^_ab_	5.36 ± 0.12^B^_c_	5.52 ± 0.09^B^_c_	5.49 ± 0.06^B^_c_	6.08 ± 0.06^B^_ab_	6.54 ± 0.09^A^_a_	6.01 ± 0.09^B^_b_	5.33 ± 0.14^B^_c_
		Cooked	32.88 ± 0.55^Y^_4_	30.40 ± 0.67^Y^_5_	28.28 ± 0.23^Y^_6_	31.32 ± 0.21^Y^_4,5_	31.68 ± 0.35^Y^_4,5_	38.38 ± 0.32^Y^_3_	40.57 ± 0.47^Y^_2_	41.84 ± 0.48^Y^_2_	45.04 ± 0.32^Y^_1_
	B	Uncooked	5.49 ± 0.11^A^_d_	6.67 ± 0.20^A^_c_	8.99 ± 0.07^A^_a_	9.12 ± 0.11^A^_a_	8.20 ± 0.21^A^_b_	9.28 ± 0.18^A^_a_	6.21 ± 0.09^A^_c_	6.67 ± 0.06^A^_c_	8.24 ± 0.15^A^_b_
		Cooked	39.95 ± 0.57^X^_4_	37.82 ± 0.23^X^_5_	37.33 ± 0.41^X^_5_	37.89 ± 0.32^X^_5_	40.31 ± 0.23^X^_3,4_	42.00 ± 0.26^X^_3_	45.08 ± 0.54^X^_2_	44.72 ± 0.28^X^_2_	48.25 ± 0.32^X^_1_
Adhesiveness (mJ)	Control	Uncooked	0.23 ± 0.03^A^_e_	0.23 ± 0.03^A^_e_	0.33 ± 0.03^A^_de_	0.37 ± 0.03^A^_de_	0.50 ± 0.06^A^_cd_	0.53 ± 0.03^A^_cd_	0.67 ± 0.07^A^_c_	0.97 ± 0.03^A^_b_	1.23 ± 0.09^A^_a_
		Cooked	0.07 ± 0.03^X^_1_	0.00 ± 0.00^X^_1_	0.00 ± 0.00^X^_1_	0.03 ± 0.03^X^_1_	0.07 ± 0.03^X^_1_	0.07 ± 0.03^X^_1_	0.07 ± 0.03^X^_1_	0.03 ± 0.03^X^_1_	0.07 ± 0.03^X^_1_
	A	Uncooked	0.02 ± 0.00^B^_a_	0.00 ± 0.00^B^_ab_	0.00 ± 0.00^B^_b_	0.00 ± 0.00^B^_ab_	0.00 ± 0.00^B^_ab_	0.00 ± 0.00^B^_ab_	0.00 ± 0.00^B^_b_	0.00 ± 0.00^B^_ab_	0.01 ± 0.00^B^_ab_
		Cooked	0.00 ± 0.00^Y^_1_	0.00 ± 0.00^X^_1_	0.00 ± 0.00^X^_1_	0.00 ± 0.00^Y^_1_	0.00 ± 0.00^Y^_1_	0.00 ± 0.00^Y^_1_	0.00 ± 0.00^Y^_1_	0.00 ± 0.00^Y^_1_	0.00 ± 0.00^Y^_1_
	B	Uncooked	0.01 ± 0.00^B^_a_	0.00 ± 0.00^B^_b_	0.00 ± 0.00^B^_b_	0.00 ± 0.00^B^_b_	0.01 ± 0.00^B^_ab_	0.01 ± 0.00^B^_ab_	0.00 ± 0.00^B^_b_	0.00 ± 0.00^B^_b_	0.00 ± 0.00^B^_ab_
		Cooked	0.00 ± 0.00^Y^_1_	0.00 ± 0.00^X^_1_	0.00 ± 0.00^X^_1_	0.00 ± 0.00^Y^_1_	0.00 ± 0.00^Y^_1_	0.00 ± 0.00^Y^_1_	0.00 ± 0.00^Y^_1_	0.00 ± 0.00^Y^_1_	0.00 ± 0.00^Y^_1_
Springiness (mm)	Control	Uncooked	6.70 ± 0.18^C^_e_	7.39 ± 0.12^C^_cd_	8.51 ± 0.23^B^_a_	8.29 ± 0.12^B^_ab_	6.78 ± 0.15^B^_de_	6.49 ± 0.07^B^_e_	7.42 ± 0.13^A^_cd_	7.41 ± 0.12^A^_cd_	7.60 ± 0.03^A^_bc_
		Cooked	7.76 ± 0.08^Z^_2,3_	8.33 ± 0.19^Z^_2_	9.49 ± 0.06^Z^_1_	7.63 ± 0.20^Z^_3_	6.97 ± 0.10^Z^_4_	5.21 ± 0.10^Z^_5_	6.41 ± 0.12^Z^_4_	6.69 ± 0.15^Z^_4_	6.57 ± 0.13^Z^_4_
	A	Uncooked	8.31 ± 0.09^B^_b_	9.74 ± 0.08^B^_a_	8.53 ± 0.14^B^_b_	6.72 ± 0.13^C^_d_	7.49 ± 0.19^B^_c_	6.15 ± 0.09^B^_e_	7.67 ± 0.10^A^_c_	7.47 ± 0.05^A^_c_	7.63 ± 0.05^A^_c_
		Cooked	11.93 ± 0.11^Y^_4_	14.39 ± 0.11^Y^_2_	15.11 ± 0.14^Y^_1_	13.93 ± 0.16^Y^_2,3_	13.35 ± 0.12^Y^_3_	11.73 ± 0.16^Y^_4,5_	11.19 ± 0.08^Y^_5_	9.69 ± 0.08^Y^_6_	8.09 ± 0.08^Y^_7_
	B	Uncooked	15.78 ± 0.56^A^_a_	16.31 ± 0.10^A^_a_	13.27 ± 0.04^A^_b_	11.55 ± 0.53^A^_c_	11.38 ± 0.25^A^_c_	7.74 ± 0.22^A^_d_	7.45 ± 0.11^A^_d_	6.91 ± 0.13^B^_d_	6.85 ± 0.05^B^_d_
		Cooked	15.22 ± 0.02^X^_4_	17.32 ± 0.06^X^_1_	16.66 ± 0.15^X^_2_	16.46 ± 0.14^X^_2,3_	16.44 ± 0.12^X^_2,3_	16.18 ± 0.02^X^_3_	16.32 ± 0.05^X^_2,3_	14.68 ± 0.04^X^_5_	14.28 ± 0.05^X^_5_
Cohesiveness	Control	Uncooked	0.42 ± 0.01^B^_b_	0.52 ± 0.01^A^_a_	0.57 ± 0.01^B^_a_	0.43 ± 0.01^C^_b_	0.34 ± 0.01^B^_b_	0.42 ± 0.00^B^_cd_	0.38 ± 0.02^B^_c_	0.31 ± 0.01^C^_de_	0.26 ± 0.01^C^_e_
		Cooked	0.35 ± 0.01^Z^_4_	0.46 ± 0.01^Z^_2,3_	0.60 ± 0.01^X^_1_	0.43 ± 0.01^Y^_3_	0.42 ± 0.01^Z^_3_	0.34 ± 0.01^Z^_4_	0.44 ± 0.02^Y^_2,3_	0.46 ± 0.02^X^_2,3_	0.51 ± 0.02^X^_2_
	A	Uncooked	0.56 ± 0.02^A^_a_	0.59 ± 0.01^A^_a_	0.56 ± 0.02^B^_a_	0.52 ± 0.01^B^_ab_	0.47 ± 0.01^A^_bc_	0.36 ± 0.01^A^_d_	0.44 ± 0.02^AB^_c_	0.40 ± 0.01^B^_cd_	0.37 ± 0.02^B^_d_
		Cooked	0.39 ± 0.01^Y^_3_	0.50 ± 0.01^Y^_2_	0.60 ± 0.01^X^_1_	0.55 ± 0.01^X^_1_	0.55 ± 0.01^Y^_1_	0.48 ± 0.01^Y^_2_	0.49 ± 0.01^XY^_2_	0.49 ± 0.00^X^_2_	0.43 ± 0.01^Y^_3_
	B	Uncooked	0.50 ± 0.01^A^_cd_	0.58 ± 0.01^A^_b_	0.68 ± 0.01^A^_a_	0.66 ± 0.02^A^_a_	0.49 ± 0.02^A^_cd_	0.46 ± 0.01^A^_d_	0.51 ± 0.01^A^_cd_	0.49 ± 0.02^A^_cd_	0.54 ± 0.01^A^_bc_
		Cooked	0.57 ± 0.01^X^_4,5_	0.64 ± 0.01^X^_1,2_	0.66 ± 0.01^Y^_1_	0.60 ± 0.01^X^_3,4_	0.61 ± 0.01^X^_2,3_	0.54 ± 0.01^X^_5,6_	0.52 ± 0.01^X^_6_	0.45 ± 0.00^X^_7_	0.46 ± 0.00^XY^_7_
Chewiness (N × mm)	Control	Uncooked	5.37 ± 0.11^C^_d_	11.29 ± 0.43^C^_a_	10.23 ± 0.08^C^_b_	8.22 ± 0.23^C^_c_	5.67 ± 0.19^C^_d_	3.75 ± 0.10^C^_e_	3.71 ± 0.13^B^_e_	3.21 ± 0.20^C^_ef_	3.07 ± 0.09^C^_f_
		Cooked	67.40 ± 0.92^Z^_3_	92.15 ± 1.29^Z^_2_	131.19 ± 1.92^Z^_1_	69.30 ± 0.07^Z^_3_	55.70 ± 2.08^Z^_4_	26.93 ± 0.75^Z^_7_	37.27 ± 1.35^Z^_6_	44.36 ± 0.42^Z^_5_	28.77 ± 0.83^Z^_7_
	A	Uncooked	19.92 ± 0.08^B^_cd_	36.59 ± 0.67^B^_a_	25.72 ± 0.33^B^_b_	19.15 ± 0.22^B^_cd_	19.45 ± 0.20^B^_cd_	13.47 ± 0.42^B^_f_	22.12 ± 1.55^A^_c_	18.14 ± 0.89^B^_de_	14.89 ± 0.51^B^_ef_
		Cooked	152.89 ± 3.53^Y^_5_	218.49 ± 1.58^Y^_2,3,4_	254.87 ± 3.39^Y^_1_	241.43 ± 7.00^Y^_1,2_	234.10 ± 8.20^Y^_1,2,3_	216.14 ± 5.87^Y^_3,4_	220.86 ± 4.41^Y^_2,3,4_	197.31 ± 3.00^Y^_4_	155.42 ± 4.78^Y^_5_
	B	Uncooked	43.81 ± 0.21^A^_d_	62.40 ± 1.36^A^_c_	82.19 ± 1.57^A^_a_	69.09 ± 2.24^A^_b_	46.21 ± 2.86^A^_d_	31.21 ± 2.66^A^_e_	22.78 ± 0.93^A^_f_	21.59 ± 0.47^A^_f_	30.46 ± 0.61^A^_e_
		Cooked	344.34 ± 4.09^X^_4_	416.92 ± 2.36^X^_1_	408.17 ± 1.46^X^_1_	372.03 ± 4.04^X^_2,3_	406.37 ± 0.80^X^_1_	369.19 ± 3.56^X^_3_	384.98 ± 2.86^X^_2_	297.50 ± 1.18^X^_6_	319.13 ± 1.92^X^_5_
Shear force (N)	Control	Uncooked	0.65 ± 0.03^B^_bc_	0.52 ± 0.03^B^_c_	0.92 ± 0.09^C^_ab_	0.98 ± 0.06^C^_a_	0.88 ± 0.06^C^_ab_	0.88 ± 0.10^C^_ab_	0.95 ± 0.03^C^_ab_	0.85 ± 0.03^C^_ab_	0.95 ± 0.07^C^_ab_
		Cooked	6.80 ± 0.12^X^_3,4_	4.81 ± 0.15^Y^_5_	6.41 ± 0.12^Z^_3,4_	6.31 ± 0.24^Z^_4_	8.70 ± 0.28^Z^_2_	7.49 ± 0.14^Z^_3_	12.19 ± 0.36^Z^_1_	12.98 ± 0.28^Y^_1_	7.49 ± 0.26^Z^_3_
	A	Uncooked	0.85 ± 0.09^B^_c_	1.05 ± 0.12^B^_c_	1.63 ± 0.03^B^_b_	1.77 ± 0.11^B^_ab_	1.77 ± 0.06^B^_ab_	1.77 ± 0.06^B^_ab_	1.77 ± 0.10^B^_ab_	1.99 ± 0.09^B^_ab_	2.12 ± 0.12^B^_a_
		Cooked	6.44 ± 0.38^X^_4_	14.84 ± 0.93^X^_1_	12.36 ± 0.39^Y^_1,2,3_	14.91 ± 0.70^Y^_1_	14.35 ± 0.12^Y^_1,2_	12.03 ± 0.34^Y^_2,3_	14.78 ± 0.79^Y^_1_	13.63 ± 0.37^Y^_1,2,3_	11.18 ± 0.40^Y^_3_
	B	Uncooked	1.73 ± 0.09^A^_d_	2.45 ± 0.17^A^_bc_	2.88 ± 0.17^A^_abc_	3.30 ± 0.09^A^_a_	3.07 ± 0.23^A^_ab_	2.65 ± 0.06^A^_abc_	2.26 ± 0.15^A^_cd_	2.42 ± 0.13^A^_bc_	3.01 ± 0.12^A^_ab_
		Cooked	6.24 ± 0.40^X^_4_	16.28 ± 0.28^X^_3_	13.93 ± 0.34^X^_3_	25.01 ± 1.59^X^_1_	20.33 ± 0.43^X^_2_	26.15 ± 0.09^X^_1_	19.71 ± 0.37^X^_2_	20.53 ± 0.42^X^_2_	15.30 ± 0.29^X^_3_
Work of shear (N × s)	Control	Uncooked	7.24 ± 0.34^C^_a_	6.28 ± 0.17^C^_b_	10.68 ± 0.38^C^_ab_	10.36 ± 1.94^B^_ab_	11.35 ± 2.01^B^_ab_	10.76 ± 1.63^C^_ab_	11.96 ± 0.32^B^_a_	9.53 ± 0.37^C^_ab_	9.15 ± 0.31^C^_ab_
		Cooked	79.58 ± 3.57^X^_3_	49.09 ± 0.53^Z^_4_	60.95 ± 1.92^Z^_4_	60.36 ± 2.02^Z^_4_	114.14 ± 3.98^Y^_2_	94.94 ± 4.13^Z^_3_	124.74 ± 4.22^Y^_2_	178.05 ± 4.89^X^_1_	82.46 ± 2.86^Y^_3_
	A	Uncooked	11.50 ± 1.37^B^_a_	11.76 ± 0.72^B^_a_	18.33 ± 1.31^B^_a_	17.18 ± 2.91^B^_a_	16.87 ± 1.78^B^_a_	16.94 ± 0.61^B^_a_	17.96 ± 1.06^A^_a_	18.25 ± 0.83^B^_a_	18.58 ± 0.84^B^_a_
		Cooked	73.16 ± 3.99^X^_4_	127.98 ± 4.96^Y^_2,3_	141.04 ± 3.73^Y^_1,2,3_	158.98 ± 5.07^Y^_1_	144.45 ± 5.38^X^_1,2,3_	148.22 ± 5.79^Y^_1,2_	143.87 ± 6.25^X^_1,2,3_	161.57 ± 8.76^X^_1_	119.40 ± 5.34^X^_3_
	B	Uncooked	22.30 ± 1.19^A^_bc_	19.09 ± 0.53^A^_c_	26.42 ± 0.63^A^_b_	33.21 ± 1.19^A^_a_	26.46 ± 1.37^A^_b_	24.37 ± 0.74^A^_bc_	21.94 ± 1.92^A^_bc_	25.15 ± 1.02^A^_b_	25.07 ± 0.59^A^_b_
		Cooked	79.33 ± 2.43^X^_6_	180.40 ± 3.63^X^_2,3_	169.33 ± 3.10^X^_2,3_	182.81 ± 4.64^X^_2_	143.38 ± 6.08^X^_4_	280.89 ± 8.11^X^_1_	156.89 ± 1.06^X^_3,4_	176.58 ± 4.89^X^_2,3_	116.35 ± 5.01^X^_5_

*Note:* Mean (*n* = 12) ± SE. A–C (↓): The difference between uncooked groups with different letters for each treatment is significant (*p* < 0.05). X–Z (↓): The difference between cooked groups with different letters for each treatment is significant (*p* < 0.05). 1–8 (→): The difference between days with different numbers for each treatment is significant (*p* < 0.05).

Compared to day‐0, the shear force values increased in all groups (*p* < 0.05). During the storage period, the control group exhibited a relatively low increase, while the enzyme‐containing groups (B > A > Control) showed a much more significant increase in shear force value (Table 7). It is known that the reason for this is the enzyme addition, which makes the textural structure tighter and harder (Uran 2013). The TPA analyses showed that using enzymes led to an increase in hardness and springiness parameters. The shear force is expected to increase in the groups due to the concentration. Moreno, Carballo, and Borderias (2008) reported that MTGase is increasing the shear force of restructured fishery products (*p* < 0.05). The work of shear values in raw fish fillets was determined as 235.22 N × s. During the storage period, group A had higher shear work values than the control, and group B had higher work of shear values than the other groups (Table 7). These results show that MTGase concentration significantly affects the work of shear. Likewise, using enzyme increases both the springiness and bonding in tissue. Moreno, Carballo, and Borderias (2008) reported that MTGase is increasing the work of shear of restructured product from hake (*p* < 0.05). The shear force (N) values of cooked burger patties were found to be similar in all groups at day‐0 (*p* > 0.05). After 3rd day, an increase in shear force was observed in the enzyme‐added groups (*p* < 0.05) (Table 7). Tellez‐Luis et al. (2002) and Çiftçi (2010) reported that the values of the shear force had been increased in enzyme‐contained groups compared to the control group (*p* < 0.05). As the initial results of the shear force, a similar situation was also seen in the work of shear (N × s) results. Because, before the cooking process, the enzyme activation period was not finished. Altan et al. (2023) noted that restructuring in low‐temperature and limited time conditions (4°C ± 1°C, 3 h) is ineffective regarding texture parameters due to a lack of enzyme activation period. In addition to that, high temperature has stopped the activation. However, by the first periodic analysis day (3rd day), the effect of MTGase was seen clearly (*p* < 0.05). Çiftçi (2010) reported that MTGase is increasing the work of shear values (*p* < 0.05).

### 3.8 Scanning Electron Microscope Image Results

**FIGURE 1:**
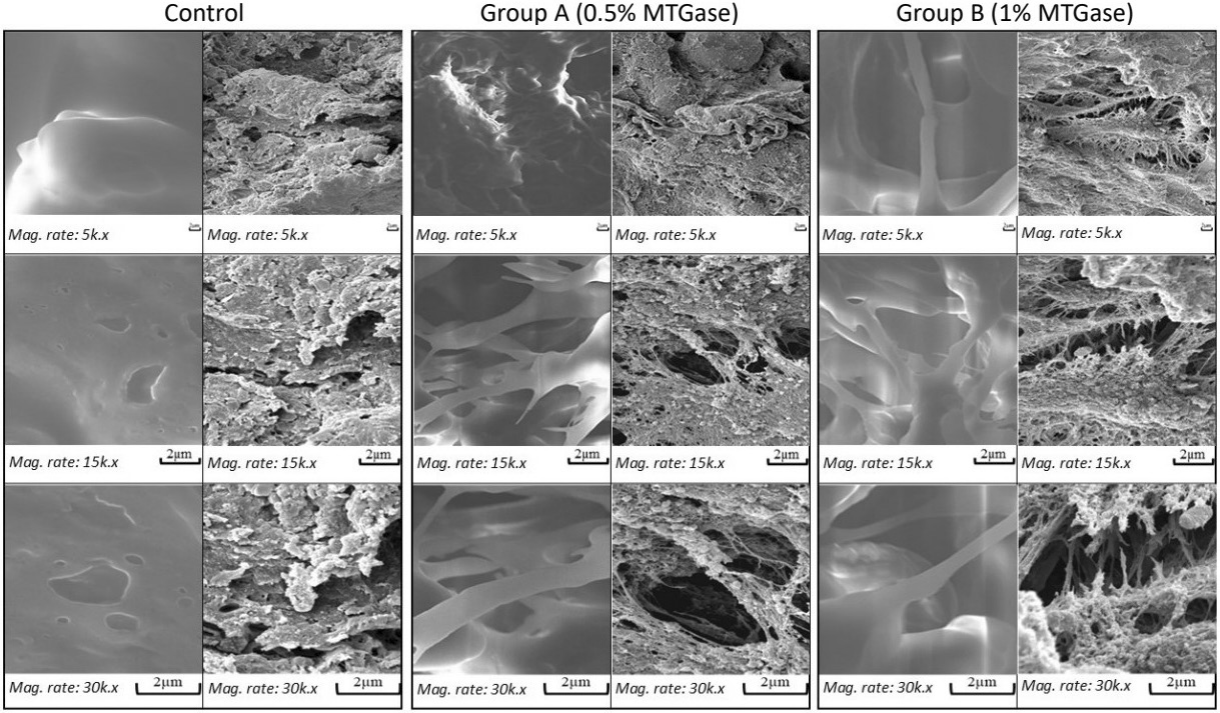
Scanning electron microscope (SEM) images.

No structural binding was found in either the first or last image of the control group (Figure 1). However, connective tissue can easily be seen in group A's first and last images. Furthermore, in group B, the connective tissue and gel network shapes were determined in a much denser and more complex structure in both the first and last imaging. These connective tissue and gel network structures were observed in the enzyme‐containing groups, and the increase in concentration is believed to be due to the formation of intermolecular ε(γ‐glutamyl)‐lysine cross‐links. Motoki and Seguro (1998) reported that using MTGase in meat products intensively forms intermolecular ε(γ‐glutamyl)‐lysine crosslinks. Cardoso et al. (2010) reported that the MTGase‐containing group had the most evident and strongest cross‐connective tissue formation. Uran (2013) found that connective tissue formation increased in chicken burgers made using TGase and that the density of G‐L compounds formed increased with increasing enzyme concentration. Gore et al. (2023) observed an increase in cross‐connective tissue in the SEM images of sausages produced from fresh Indian major carp (*Labeo rohita*) fish in the groups using MTGase, and they reported that the amount of cross‐connective tissue increased with the increase in enzyme concentration. The researchers' findings are consistent with our findings.

## 4 Conclusions

The results of this research showed that MTGase enzyme exhibited protective/improving effects on the textural, physicochemical, and nutritional parameters of trout burger patties compared to the control group. When all microbiological analyses were evaluated together, MTGase‐containing groups obtained better results in inhibiting microbial fauna. Moreover, in proportion to the increase in enzyme concentration, TMAB inhibition was enhanced, and textural properties improved in both cooked and raw samples. The cross‐linked tissue formations observed in SEM images correlate strongly with the textural findings. In conclusion, trout burger patties containing both enzyme ratios can be safely consumed; however, considering the advantages of using a higher enzyme concentration (1%), particularly in terms of textural properties, it can be posited that the enzyme ratio of 1% is more convenient for commercial production.

## Author Contributions

**Can Okan Altan:** formal analysis (equal), supervision (equal), writing – review and editing (equal). **Hülya Turan:** supervision (equal), writing – review and editing (equal). **Demet Kocatepe:** formal analysis (equal), writing – review and editing (equal). **İrfan Keskin:** formal analysis (equal). **Bayram Köstekli:** formal analysis (equal).

## Conflicts of Interest

The authors declare no conflicts of interest.

## Data Availability

The data that support the findings of this study are available on request from the corresponding author. The data are not publicly available due to privacy or ethical restrictions.
